# Effects of modafinil and caffeine on night-time vigilance of air force crewmembers: A randomized controlled trial

**DOI:** 10.1177/02698811221142568

**Published:** 2022-12-14

**Authors:** Yara Q. Wingelaar-Jagt, Charelle Bottenheft, Wim J. Riedel, Johannes G. Ramaekers

**Affiliations:** 1 Center for Man in Aviation, Royal Netherlands Air Force, Utrecht, The Netherlands; 2 Department of Neuropsychology and Psychopharmacology, Faculty of Psychology and Neuroscience, Maastricht University, Maastricht, The Netherlands; 3 TNO Netherlands Organization for Applied Scientific Research, Utrecht, The Netherlands

**Keywords:** Aviation, fatigue, shift work, sleep, wakefulness-promoting agents

## Abstract

**Background:**

Fatigue remains an important factor in major aviation accidents. Stimulants may counteract fatigue’s adverse effects, with modafinil as a promising alternative to caffeine. However, the effect of a single dose of modafinil after a limited period of sleep deprivation remains unknown.

**Aims:**

This study aims to determine the effect of 200 mg modafinil on vigilance during a limited period of sleep deprivation compared to 300 mg caffeine and placebo.

**Methods:**

Thirty-two volunteers of the Royal Netherlands Air Force (RNLAF) were double-blindly administered modafinil, caffeine, and placebo on three non-consecutive trial days after being awake for median 17 h. Afterwards, subjects completed six series of the Vigilance and Tracking test (VigTrack), psychomotor vigilance task (PVT), and Stanford Sleepiness Scale (SSS), yielding six primary endpoints.

**Results:**

This study revealed statistically significant effects of caffeine and modafinil compared with placebo on all endpoints, except for VigTrack mean tracking error. PVT results were less impaired 2 h after administration, followed by VigTrack parameters and SSS scores 2 h thereafter. Compared with caffeine, modafinil significantly improved PVT and SSS scores at 8 h after administration.

**Conclusions:**

The present study demonstrates that 200 mg modafinil and 300 mg caffeine significantly decrease the effects of a limited period of sleep deprivation on vigilance compared with placebo. Although PVT parameters already improved 2 h after administration, the most notable effects occurred 2–4 h later. Modafinil seems to be effective longer than caffeine, which is consistent with its longer half-life.

## Introduction

In 2010, for the first time in an air crash investigation, a recording of snoring was identified on a cockpit voice recorder (Court of Inquiry India, 2010). This cockpit voice recorder belonged to Air India Express Flight 812, which crashed, killing 158 of the 166 persons onboard. The recording indicated that the captain had been asleep for more than 90 min of the 2 h flight. Residual sleepiness and impaired judgment were identified as contributing factors in this accident. The captain’s fatigue was suggested to be due to flying during the Window of Circadian Low (WOCL), the period of the circadian cycle when fatigue and sleepiness are greatest and people are least able to perform mental or physical work (Valdez, 2019).

This is not an isolated instance of an aviation accident being attributed to fatigue. In the last two decades, fatigue has been identified as the probable cause of 21–24% of major aviation accidents, both in civil and military aviation (Caldwell, 2012; Gaines et al., 2020; Marcus and Rosekind, 2017). As stated in the International Civil Aviation Organization’s (ICAO) definition of fatigue, fatigue can impair one’s performance: “*A physiological state of reduced mental or physical performance capability resulting from sleep loss, extended wakefulness, circadian phase, and/or workload (mental and/or physical activity) that can impair a person’s alertness and ability to perform safety related operational duties*” (ICAO, 2020).

This definition identifies several possible causes of fatigue, with sleep loss probably being the most notable. The optimal method of avoiding fatigue is to have sufficient (night-time) sleep. However, this is often difficult to achieve, especially during military deployments, because sleep in the field is often of poorer quality and shorter duration than sleep at home (Kelley et al., 2018). Moreover, performing military operations at night may be tactically necessary, thereby disrupting the normal sleep pattern. This, combined with possible interfering transient factors like noise or heat, may lead to irregular sleep during deployment, which may cause fatigue. Additionally, the deployment itself, with the mission and potential threats, may induce stress, which may also contribute to fatigue. This is particularly problematic at the end of flight missions because the landing phase is a risk factor for the occurrence of aviation accidents (European Union Aviation Safety Agency [EASA], 2020). Also, when performing night-time operations, pilots might be forced to fly during circadian phases dedicated for sleep, like the WOCL, when levels of attention are at their lowest, additionally increasing the chance of incidents.

Regulations limiting flight times and suggesting optimal rosters have been implemented by aviation authorities (EASA, 2014; Federal Aviation Administration, 2012). Although these cannot completely prevent fatigue, they provide a framework to manage fatigue (Wingelaar-Jagt et al., 2021). However, the introduction of these regulations in the Royal Netherlands Air Force (RNLAF) is complicated by the variety of aircrafts available and the types of operations performed. Additionally, there is the possibility of deviating from these regulations in the case of operational necessity. These circumstances make it impossible to solely rely on these regulations to manage fatigue and its associated risks. Other countermeasures are therefore needed to enhance the fitness of pilots to fly under these circumstances. Currently, the RNLAF allows its pilots to use certain hypnotics to get sufficient sleep prior to flight operations (Military Aviation Authority, 2021).

Depending on the scenario, an alternative option is to prescribe stimulants, that is, medications that increase vigilance and reduce fatigue. Although caffeine is widely available, both in pills and beverages, aircrew members have reported that caffeine supplements are ineffective, which might be due to the high daily caffeine consumption of many (Chou et al., 1985; Nehlig, 2018). Additionally, caffeine has a relative short half-life of 4–6 h, which might be less favorable when longer periods of vigilance are needed, for example, during long night-time operations.

Modafinil is a relatively new wakefulness-promoting drug that has been approved as an agent to counter fatigue by the air forces of Singapore, the United States, India, and France (Ooi et al., 2019). Although its exact mechanism of action remains undetermined, it is thought to exert a stimulating effect by altering the levels of several neurotransmitters, including serotonin, noradrenalin, dopamine, and gamma-aminobutyric acid (Battleday and Brem, 2015; Kim, 2012). It has a longer *T*_max_ (2–4 h) and *T*_1/2_ (12–15 h) than caffeine (30–120 min and 4–6 h, respectively) (Robertson and Hellriegel, 2003; Wingelaar-Jagt et al., 2021). Evaluations of the efficacies of pharmaceutical agents showed that modafinil is a promising fatigue countermeasure. However, this was mostly studied after longer periods of sleep deprivation, sometimes lasting >40 h (Killgore et al., 2006, 2008; Wesensten et al., 2002, 2004, 2005). By contrast, studies evaluating the effect of modafinil after shorter periods of sleep deprivation used multiple doses (Caldwell et al., 2000, 2004; Estrada et al., 2012). The effect of a single dose of modafinil after a similar limited period of wakefulness (e.g., 24 h) has not been studied extensively. This timeframe is particularly interesting for military aviation because this scenario is most likely during operational missions.

The present study aimed to determine the effect of a single dose of modafinil (200 mg) on vigilance during a limited period of sleep deprivation compared with those of placebo and a single dose of caffeine (300 mg). The period of sleep deprivation was 24 h, and special attention was paid to the level of vigilance during the WOCL. We expected both modafinil and caffeine to counteract the effects of fatigue on vigilance compared with placebo, with the beneficial effects of caffeine occurring earlier than those of modafinil due to the difference in *T*_max_.

## Materials and methods

### Participants

This randomized, double-blind, crossover, active- and placebo-controlled clinical trial was conducted at the Center for Man in Aviation, RNLAF (Soesterberg, the Netherlands) and adhered to the principles of the Declaration of Helsinki, the International Council on Harmonization, and the Good Clinical Practice guidelines. The protocol was approved by the Medical Ethical Committee Brabant (reference: NL62145.028.17/P1749) and the Surgeon General of the Ministry of Defence. The study was registered in the Dutch Trial Register (No. NTR6922) and EU Clinical Trials Register (No. 2017-002288-16).

Healthy employees of the RNLAF aged between 18 and 60 years were eligible for inclusion. Eligible participants were fit to fly according to the RNLAF Military Aviation Regulations or European Aviation Regulations (European Aviation Safety Authority [EASA], 2011; Military Aviation Authority, 2020). Exclusion criteria were mainly based on possible side effects or interactions of one or both medicines, for example, pregnancy or breastfeeding, the use of medication that is metabolized through CYP3A4/5, CYP2C19, or CYP2C9, and/or a history of psychiatric illness including sleep disorders, or the use of psychoactive drugs.

After being informed, both verbally and in writing, about the aims, consequences, and constraints of the study, all participants gave written consent. This informed consent was voluntary and could be retracted at any time without any consequences. According to (inter)national privacy regulations, no study data were included in the medical files of the participants.

This study included 32 subjects, two of whom only completed two of the three test days due to operational reasons. Both subjects missed the caffeine administration; per protocol their test results were included in the analysis. The subjects were aged between 25 and 59 years (mean: 35 years; standard deviation: 10 years). Five (16%) of the 32 subjects were female and 21 (66%) of all subjects were pilots. On the test days, the median waking time of the subjects was 07:00 AM, meaning that at medication administration, the subjects had a median period of wakefulness of 17 h (range: 15.5–20.0 h, (interquartile range) IQR: 16.5–18.0 h).

### Materials

The Vigilance and Tracking test (VigTrack) is a dual-task that measures vigilance performance under the continuous load of a compensatory tracking task. The test has been used in various studies and is sensitive for measuring vigilance and alertness (Simons, 2017; Valk and Simons, 2009). During the tracking task, participants had to steer a blue dot using a joystick such that it remained below a red dot in the center of the display. The blue dot is programmed to move continuously from the center of the display. While tracking, participants had to perform the vigilance task. Inside the red dot, a black square alternated with a diamond, once per second. At random intervals, a hexagon was presented. When this occurred, participants had to press an additional key on the joystick. The duration of this test was 10 min, and primary endpoints included root mean square tracking error, percentage omissions, and mean reaction time.

The psychomotor vigilance task (PVT) measures the speed with which subjects respond to a red stimulus and is used to assess the vigilance of subjects (Basner and Dinges, 2011). The interstimulus interval, defined as the period between the last response and the appearance of the next stimulus, varies randomly from 2 to 10 s. The duration of this test was 10 min, and primary endpoints included reciprocal (1/mean) reaction time and lapses. Lapses (errors of omission) were defined as RTs ⩾ 500 ms.

At the start of every trial day, a familiarization session of 5 min per task was scheduled for all subjects to avoid practice bias during the actual measurements.

The Stanford Sleepiness Scale (SSS) was used to subjectively assess the degree of sleepiness in subjects during the test days (Hoddes et al., 1973). This subjective rating scale is sensitive to detect any significant increase in sleepiness or fatigue, and it is highly correlated with flying performance and the threshold of information-processing speed during periods of intense fatigue (Perelli, 1980).

Blood samples were taken four times throughout the night to determine modafinil and caffeine blood levels (at *T* = 0, *T* = +3, *T* = +6 and *T* = +8). These samples were taken by qualified medical personnel in concordance with Dutch quality and safety standards and were analyzed by an external, qualified diagnostic laboratory.

After each test day, subjects were asked to complete sleep questionnaires about their sleep on the day and night immediately following the test day and night. After the last test day, the participants were asked to report which medication they believed they had been administered on which night.

### Design

This trial had a within-subjects 3 × 7 design: treatment (modafinil, caffeine, placebo) × time (*T* = −6, *T* = 0, *T* = +1, *T* = +2, *T* = +3, *T* = +4, *T* = +6, *T* = +8). The entire study consisted of three non-consecutive trial days for every participant during which modafinil, caffeine, and placebo capsules were each administered once just after midnight (see Table 1). The dose of modafinil was 200 mg, which is regarded as an effective dose as a countermeasure for fatigue in military aviation (Caldwell et al., 2000, 2009). The dose of caffeine (300 mg) was the usual dose administered to RNLAF aviators nowadays; it is considered a medium-range but effective dose (Caldwell et al., 2009; Lohi et al., 2007).

**Table 1.** Overview of study design and data collection. All study days were identical, the only difference being the medication administered (modafinil 200 mg, caffeine 300 mg, or placebo).

Timing	Activity
The 3 days before every test day	Sleep diary
	Caffeine log
4:30 PM	Vital parameters
	SSS
	Familiarization with PVT and VigTrack
6:00 PM	Baseline block (*T* = −6)
	SSS
	Assessment of VigTrack and PVT
Midnight	Second baseline block (*T* = 0)
	Vital parameters
	SSS
	Assessment of VigTrack and PVT
	Test medication administration
1:00 AM	First test block (*T* = +1)
	SSS
	Assessment of VigTrack and PVT
2:00 AM	Second test block (*T* = +2)
	Vital parameters
	SSS
	Assessment of VigTrack and PVT
3:00 AM	Third test block (*T* = +3)
	SSS
	Assessment of VigTrack and PVT
4:00 AM	Fourth test block (*T* = +4)
	SSS
	Assessment of VigTrack and PVT
6:00 AM	Fifth test block (*T* = +6)
	SSS
	Assessment of VigTrack and PVT
8:00 AM	Sixth test block (*T* = +8)
	Vital parameters
	SSS
	Assessment of VigTrack and PVT
Outtake	Sleep questionnaires

A wash-out period of at least 7 days was implemented to ensure that the investigational products were completely eliminated and would not interfere on subsequent trial days.

The study was double-blinded as both the subjects and investigators were unaware of the treatment given on test days. The order of the treatments for each individual subject (placebo, caffeine, or modafinil) was based on a computer-generated randomization schedule organized and monitored by an external statistician. Randomization was performed using all possible (six) treatment sequences to ensure balance for carryover effects, that is, improving skills or learning bias on the test battery. For every test day the researchers received a treatment kit from the pharmacist. The treatment kits were labeled with the subject number and the test day and contained identical capsules.

### Procedure

One week prior to the start of every trial day, participants remained within the time zone of the research center (GMT + 1, daylight-saving GMT + 2) to prevent jetlag, which might confound the test results. During the trial days, no strenuous physical exercise (including sports) or sleeping was allowed, and participants kept a log of their activities and caffeine intake. They were able to consume their normal amount of caffeine-based products until 5:00 PM. To avoid interference from caffeine with vigilance, the participants ceased their consumption of caffeine products from 5:00 PM on the test days.

On three consecutive days before each test day, the participants recorded their fatigue level, sleep hygiene and habits, and daily caffeine intake in a journal. These results will be analyzed and published separately. Vital signs (temperature, blood pressure, and pulse) were collected four times during each test day, two times prior to medication administration, and 2 and 8 h after administration (see Table 1). Additionally, on every test day, female subjects were tested for pregnancy and all participants were asked if they had taken any concomitant medication or unauthorized medications during the past 3 days.

Adverse events were recorded throughout the study and at every visit after screening. Subjects were asked about any adverse events multiple times during the trial days.

### Statistical analysis

Sample size calculations were performed with G*Power (Faul et al., 2007). The assumed means and standard deviations of VigTrack were used to obtain the effect size (*d*) for sample size analysis (Klopping et al., 2005). Two-way testing using a repeated-measures analysis of variance (ANOVA) within groups, with α = 0.05, β = 0.8, and the aforementioned effect size (*d*), required a minimum of *n* = 18 to show the effects of caffeine and modafinil. However, to compensate for dropouts and sample failures, 30 subjects were included. Test results were included if subjects completed at least 2 full days of testing (i.e., results of subjects that completed only one test day were excluded because within-group analyses could not be performed).

Statistical analyses were performed using IBM SPSS software version 27.0. A factorial repeated-measures ANOVA was conducted to analyze the main and interaction effects of time and treatment on the VigTrack and PVT parameters. When the average test revealed a significant overall difference, pairwise comparisons were conducted to analyze the difference between treatments. These consisted of paired comparisons between scores and between treatment conditions for all separate test sessions (least significant difference). SSS scores were analyzed by nonparametric tests (Friedman test for repeated measures and Wilcoxon matched-pairs signed-rank test for pairwise comparisons). The placebo group was included for reference purposes.

For all primary endpoints, the change from baseline, defined as the difference between the measure before drug intake (*T* = −6) and at each timepoint thereafter (*T* = 0 to *T* = +8), was calculated. Mauchly’s test was performed to test if the assumption of sphericity had been violated for the different parameters. If this was the case, the degrees of freedom were corrected using Huynh–Feldt estimates of sphericity. A *p*-value of <0.05 was considered statistically significant.

## Results

No adverse events were encountered during the study. The subjects’ vital signs were unaffected by drug administration. The study ended according to protocol.

After the last test day, the participants were asked to guess which medication they had taken on which night. Of the 94 guesses, 54 (57%) were correct. Of the 32 times, modafinil was administered, five (16%) subjects believed they had taken placebo, eight (25%) thought they had taken caffeine, and 19 (59%) guessed correctly. Of the 30 times caffeine was administered, six (20%) subjects thought they had taken placebo, seven (23%) believed they had taken modafinil, one (3%) did not know, and 16 (53%) identified the medication correctly. Of the 32 times placebo was administered, five (16%) subjects assumed they had taken modafinil, seven (22%) suspected they had taken caffeine, one (3%) did not know, and 19 (59%) identified placebo correctly. These results suggest that there was no unblinding of subjects during the study.

Plasma concentrations of modafinil and caffeine can be found in Table 2.

**Table 2.** Plasma concentrations of caffeine (μg/ml) and modafinil (mg/L).

Time	Caffeine	Modafinil
	Median (IQR)	Median (IQR)
*T* = 0 (0:00 AM)	1.30 (0.43–3.45)	0.00 (0.00–0.00)
*T* = +3 (3:00 AM)	6.65 (5.03–8.13)	4.50 (3.33–7.00)
*T* = +6 (6:00 AM)	4.30 (3.10–7.25)	5.20 (3.30–6.40)
*T* = +8 (8:00 AM)	4.25 (2.68–5.38)	4.50 (2.68–5.43)

IQR: interquartile range.

After checking for outliers in the data with boxplots, two participants were removed from the analysis of the VigTrack parameters. These participants showed extreme values for all the VigTrack parameters, likely because they may have not understood the task properly. No outliers were identified when analyzing other parameters.

**Figure 1.**
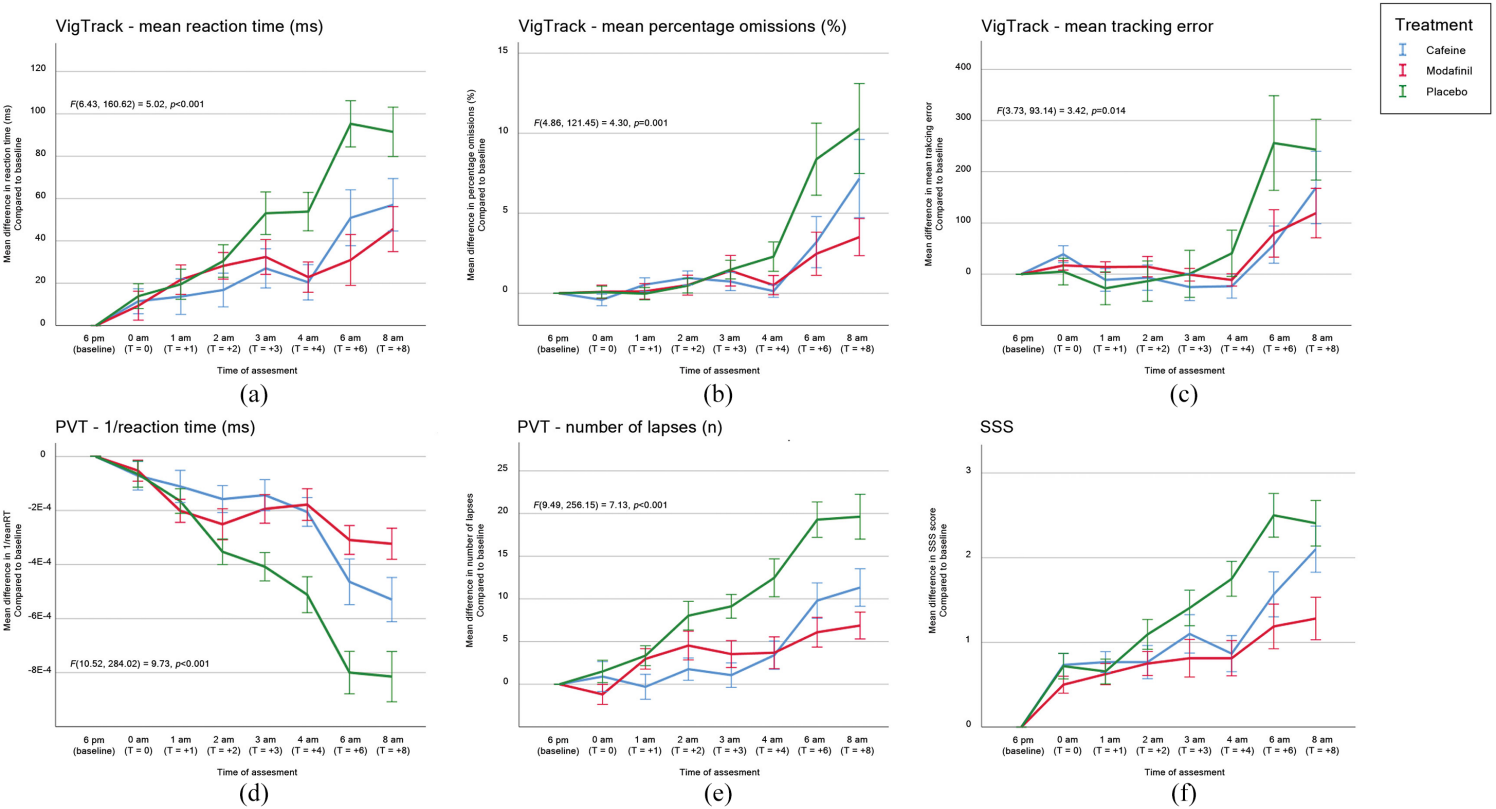
Mean differences in parameters compared to baseline per treatment and time of assessment: (a) VigTrack–mean reaction time, (b) VigTrack–mean percentage omissions, (c) VigTrack–mean tracking error, (d) PVT–1/reaction time, (e) PVT–number of lapses, and (f) SSS.

The results of Mauchly’s test and subsequent correction of the degrees of freedom are provided in the appendix. Test results for all primary endpoints are displayed in Figure 1 and described in the following paragraphs, with the *p-*values of the pairwise comparisons summarized in Supplemental Table A.1 data.

### VigTrack–mean reaction time

There was a significant main effect of treatment on mean reaction time (*F*(2, 50) = 5.71, *p* = 0.006). Post-hoc pairwise comparisons revealed that mean reaction time in seconds was significantly lower for both modafinil and caffeine than for placebo (*p* = 0.005 and *p* = 0.006, respectively).

There was a significant main effect of time of assessment on mean reaction time (*F*(2.32, 69.31) = 23.57, *p* *<* 0.001). There was also a significant interaction effect between time of assessment and treatment on mean reaction time (*F*(6.43, 160.62) = 5.02, *p* < 0.001). This indicates that the treatment had different effects on mean reaction time depending on the time of assessment.

Post-hoc pairwise comparisons revealed that performance was significantly less impaired with both modafinil and caffeine than with placebo during assessment at *T* = +4, *T* = +6, and *T* = +8.

### VigTrack—mean percentage omissions

There was a significant effect of treatment on percentage omissions (*F*(2,50) = 3.31, *p* = 0.045). Post-hoc tests revealed that percentage omissions were significantly lower for modafinil than for placebo (*p* = 0.018).

There was a significant main effect of time of assessment on percentage omissions (*F*(1.55, 38.65) = 9.57, *p* = 0.001). There was also a significant interaction effect between time of assessment and treatment on percentage omissions (*F*(4.86, 121.45) = 4.30, *p* = 0.001). This indicates that the treatment had different effects on percentage omissions depending on the time of assessment.

Post-hoc pairwise comparisons revealed that performance was less impaired with modafinil than with placebo during assessment at *T* = +6 and *T* = +8. Performance was less impaired with caffeine than with placebo during assessment at *T* = +6, and *T* = +8.

### VigTrack—mean tracking error

There was no significant main effect of treatment on mean tracking error (*F*(1.34, 33.49) = 0.86, *p* = 0.392). There was a significant main effect of time of assessment on mean tracking error (*F*(2.24, 55.88) = 9.26, *p* *<* 0.001). There was also a significant interaction effect between time of assessment and treatment on mean tracking error (*F*(3.73, 93.14) = 3.42, *p* = 0.014). This indicates that the treatment had different effects on mean tracking error depending on the time of assessment.

Post-hoc pairwise comparisons revealed that performance was less impaired with modafinil than with placebo during assessment at *T* = +6 and *T* = +8. There were no significant differences between caffeine and placebo.

### PVT—1/reaction time

There was a significant main effect of treatment on 1/mean reaction time (*F*(2.00, 54.00) = 11.50, *p* *<* 0.001). Post-hoc tests revealed that 1/mean reaction time was significantly higher for both modafinil and caffeine than for placebo (*p* < 0.001 and *p* = 0.003, respectively).

There was a significant main effect of time of assessment on 1/mean reaction time (*F*(4.65, 125.54) = 44.86, *p* *<* 0.001). There was also a significant interaction effect between time of assessment and treatment on 1/mean reaction time (*F*(10.52, 284.02) = 9.73, *p* *<* 0.001). This indicates that the treatment had different effects on 1/mean reaction time depending on the time of assessment.

Post-hoc pairwise comparisons revealed that performance was less impaired with both caffeine and modafinil than with placebo during assessment at *T* = +2, *T* = +3, *T* = +4, *T* = +6, and *T* = +8. Additionally, performance was significantly less impaired with modafinil than with caffeine during assessment at *T* = +6 and *T* = +8.

### PVT—number of lapses

There was a significant main effect of treatment on number of lapses (*F*(2, 54) = 14.15, *p* *<* 0.001). Post-hoc tests revealed that the number of lapses was significantly lower for both modafinil and caffeine than for placebo (*p* < 0.001 and *p* = 0.001, respectively).

There was a significant main effect of time of assessment on number of lapses (*F*(3.83, 131.35) = 28.53, *p* *<* 0.001). There was also a significant interaction effect between time of assessment and treatment on number of lapses (*F*(9.49, 256.15) = 7.13, *p* *<* 0.001). This indicates that the treatment had different effects on number of lapses depending on the time of assessment.

Post-hoc pairwise comparisons revealed that performance was less impaired with caffeine than with placebo during assessment at *T* = +2, *T* = +3, *T* = +4, *T* = +6, and *T* = +8. Performance was less impaired with modafinil than with placebo during assessment at *T* = +2, *T* = +3, *T* = +4, *T* = +6, and *T* = +8. Additionally, performance was significantly less impaired with modafinil than with caffeine during assessment at *T* = +8.

### SSS

The Friedman test showed that SSS scores significantly differed between the treatments during assessment at *T* = +4 (χ^2^(2) = 10.63, *p* = 0.005), *T* = +6 (χ^2^(2) = 9.31, *p* = 0.009), and *T* = +8 (χ^2^(2) = 11.08, *p* = 0.004). To investigate where the differences occurred, separate Wilcoxon signed-rank tests were conducted. Wilcoxon matched-pairs analysis revealed significantly lower SSS scores for modafinil than for placebo during assessment at, *T* = +4, *T* = +6, and *T* = +8. SSS scores were significantly lower for caffeine than for placebo during assessment at *T* = +4 and *T* = +6. SSS scores were lower for modafinil than for caffeine during assessment at *T* = +8.

## Discussion

The present study demonstrates that 200 mg modafinil and 300 mg caffeine significantly improve vigilance compared with placebo during an extended period of continuous wakefulness (mean 17.3 h), including the WOCL, without causing side effects. The most notable effects occurred in the early morning (between 4:00 and 6:00 AM), although PVT parameters improved as early as 2 h after administration. The increase in vigilance with both modafinil and caffeine was confirmed by the PVT, VigTrack, and SSS parameters. To our knowledge, this is the first randomized placebo-controlled trial to demonstrate the beneficial effects of these pharmaceutical agents after limited sleep deprivation.

Our findings are in line with the literature, although previous studies investigated the effects of caffeine and modafinil after longer periods of sustained wakefulness (Killgore et al., 2008; Wesensten et al., 2005; Wingelaar-Jagt et al., 2021). Modafinil sustains flight performance and mood state during continuous wakefulness when tested during simulated or in-flight operations, while the results for caffeine were mixed and inconclusive in these studies (Ehlert and Wilson, 2021). The effects of modafinil and caffeine appear almost simultaneously, despite their significantly different *T*_max_ (30–120 min for caffeine and 2–4 h for modafinil) (Institute of Medicine (US) Committee on Military Nutrition Research, 2001; Robertson and Hellriegel, 2003). Performance was less impaired with both modafinil and caffeine than with placebo for all PVT parameters from 2 h after administration. Additionally, from *T* = +4, subjects had faster reaction times in the VigTrack test and lower SSS scores. This was followed by improvements of the remaining study parameters 6 h after administration (except for VigTrack mean tracking error for caffeine). This is consistent with *T*_max_ of modafinil (2–4 h). However, considering that the *T*_max_ of caffeine is 30–120 min, the effects of caffeine were expected to be visible earlier than the 2–6 h after administration as observed in this study. On the other hand, in a previous study in which caffeine was given to counteract the effects of temazepam, it improved performance and alertness after 1.5 h, which is comparable to this study (Klopping et al., 2005). An explanation for the delayed onset of effects of caffeine administration in this study may be the relatively early timing of medication intake (12:00 AM). The median regular bedtime of the subjects was 11:05 PM, that is, at the moment of medication administration they were awake 0.9 h longer than normally. Likewise, at medication administration the subjects had been awake for a median of 17 h. This is slightly longer than the 16 h during which well-rested individuals can maintain high levels of alertness and performance (Van Dongen et al., 2003). Additionally, the WOCL starts at 2:00 AM, initiating the period in which humans are less effective and levels of attention are lowest. This could explain the increase in effects seen after 2:00 AM and the delayed start of the effects of caffeine in this study.

At *T* = +8, the modafinil test group showed less impaired performance in all parameters, while caffeine showed no effect on the SSS and VigTrack mean tracking error. The PVT parameters and SSS showed an increase in vigilance with modafinil compared with caffeine during assessment at *T* = +8, which is in line with the longer *T*_max_ (2–4 h) and *T*_1/2_ (12–15 h) of modafinil than of caffeine (30–120 min and 4–6 h, respectively) (Klopping et al., 2005; Robertson and Hellriegel, 2003). This explains the decrease in performance improvements with caffeine, but not with modafinil, starting at *T* = +6. Due to its long half-life, modafinil likely continues to be effective for hours after the end of the test period used in this study. This was shown in previous studies, in which the effects of modafinil remained noticeable after 10–12 h (Killgore et al., 2008; Wesensten et al., 2005). If the measurements had been continued after *T* = +8, it might have been possible to identify the duration of the effects of caffeine and modafinil on performance and vigilance. However, the test period used in this study is relevant for the RNLAF because it is congruent with common operational missions. RNLAF pilots are not kept awake for more than 24 h. While, it is possible that after being awake for a normal day (16–17 h), they are asked to perform a mission at the moment when their performance starts to decrease due to operational necessity (Van Dongen et al., 2003). Even with this restricted test period, it is clear that modafinil and caffeine have different periods of effectiveness. Thus, it is prudent to consider which stimulant offers the desired period of performance improvements.

Subjects did not always correctly identify which medication they had taken. In slightly more than half of instances, they were correct. Approximately 25% of subjects mistook modafinil for caffeine or vice versa, and 16–20% of subjects mistook modafinil or caffeine for placebo. The effects of modafinil and caffeine were more pronounced when interpreting the PVT scores than VigTrack parameters. This may be explained by the difference in the difficulty of the tasks. The PVT is a relatively simple task that is more sensitive to (feelings of) fatigue than VigTrack. By contrast, VigTrack is a more complicated and challenging test that may induce more motivation to perform and stay awake. Additionally, although both tests are sensitive for measuring vigilance and alertness, they are not comparable to the work load or complexity of tasks demanded of pilots in the cockpit. Performance improvements are more pronounced in simulator studies than in in-flight testing (Ehlert and Wilson, 2021). Potential explanations are the more demanding conditions and potentially increased arousal of pilots in-flight (Caldwell and Roberts, 2000). This could also be relevant to the present study, which was performed in a controlled laboratory environment and used relatively simple tasks. Therefore, our findings should be carefully extrapolated to real-life scenarios. Future studies are required to determine the effectiveness of stimulants during actual air operations.

Additionally, the effects found in this study may have been biased by the subjects’ level of caffeine consumption. Although the subjects ceased all caffeine consumption from 5:00 PM on the test days, the effects of their habitual caffeine intake may have still influenced their performance. Supplementary analysis is needed to determine the effect of daily caffeine consumption on the effects of stimulants during periods of sleep deprivation, and it may help to personalize stimulant use in pilots. Conversely, minor aberrations in the manufacturing process could have affected the results. While we believe these to be negligible, as the manufacturer complied with national legislation and good clinical practice, we cannot rule this out.

Caffeine plasma concentrations measurements are in line with its pharmacokinetic characteristics (*T*_max_ 30–120 min and *T*_1/2_ 4–6 h), even though the peak plasma concentration was probably before *T* = +3. The measured caffeine plasma concentrations from *T* = +3 on are in the therapeutic range of 4 to 10 μg/ml (Schulz and Schmoldt, 2003). The height of the modafinil peak plasma concentration in the present study is comparable to literature, even though in other studies the peak concentration was reached earlier after administration (1.5–2 h) (Darwish et al., 2009; Robertson and Hellriegel, 2003). Furthermore, when comparing the modafinil plasma concentrations with its pharmacokinetic characteristics (*T*_max_ 2–4 h and *T*_1/2_ 12–15 h) one would have expected the peak plasma concentration to occur earlier than at *T* = +6. A possible explanation is that the true peak plasma concentration was between *T* = +3 and *T* = +6 and was missed due to the low number of blood samples. Although this limited number of blood samples is a limitation of this study, with the 6 and 8 h follow-up time, we were able to provide details of serum concentrations relatively long after administration.

Moreover, sleep-related factors were not considered in this study. Sleep deprivation and also an extended period of wakefulness may negatively affect performance (Wingelaar-Jagt et al., 2021). To best reflect circumstances of operational military aviation, the participants were not imposed with bedtimes or waking times; therefore, the time since the last sleeping period and the duration of that sleeping period differed between subjects. These differences may have caused variation in performance during the test periods. It would be insightful from an academic perspective to investigate how much of an influence this actually constitutes. However, due to its crossover design, we do not believe this affected the results of our study. Additionally, the results presented in this study reflect in vivo benefit from modafinil and caffeine, and therefore they provide operational relevant data for military aviation. Furthermore, the effects of modafinil and caffeine on subsequent sleep periods were not considered in this analysis. The literature is ambiguous regarding the effects of modafinil on recovery sleep. One study reported that recovery sleep 16 h after modafinil administration was of a lesser quality and quantity (Estrada et al., 2012), while other studies showed that recovery sleep was unaffected (Killgore et al., 2008; Walsh et al., 2004).

In conclusion, both modafinil and caffeine improved vigilance and performance based on the PVT and VigTrack, and resulted in a lower level of reported sleepiness after a limited period of sleep deprivation. Modafinil was effective for longer than caffeine, which is consistent with its longer half-life. The effects of both modafinil and caffeine were noticeable approximately 2 h after drug administration. The delayed effect of caffeine in comparison with its short *T*_max_ of 30–120 min may be due to the relatively short period of wakefulness and subsequent start of the WOCL. Stimulants may play an important role in military aviation, especially in situations where pilots are already fatigued but operational necessity requires them to continue their mission. Therefore, it is paramount to be able to choose the optimal stimulant for the situation. Additional research evaluating the effects of modafinil and caffeine on in-flight performance, the effects of previous caffeine administration and extent of sleep deprivation, and the effects of modafinil on recovery sleep is needed to provide an evidence-based basis for this choice. Lastly, as our data suggest that modafinil continues to positively affect performance 8 h after administration, future studies could explore this. Aviation is not the only industry in which peak performance is demanded during night-time or after periods of sleep deprivation. Therefore, these results may also prove to be relevant for employees and employers in other fields, such as healthcare and logistics.

## Significance statement

Fatigue remains an important safety risk in aviation. Stimulants, like modafinil and caffeine, counteract fatigue’s adverse effects on vigilance and performance, and each has its own characteristics and optimal timeframe. Stimulants may be of particular importance in situations where pilots are already fatigued, but operational necessity requires them to continue their mission. Aviation is not the only industry in which peak performance is demanded during night-time or after periods of sleep deprivation. Therefore, it is paramount to better understand these stimulants in order to select the optimal stimulant for each situation. This may improve safety not only in aviation, but also in other fields, such as healthcare and logistics.

## Supplemental Material

sj-doc-1-jop-10.1177_02698811221142568 – Supplemental material for Effects of modafinil and caffeine on night-time vigilance of air force crewmembers: A randomized controlled trial Click here for additional data file. Supplemental material, sj-doc-1-jop-10.1177_02698811221142568 for Effects of modafinil and caffeine on night-time vigilance of air force crewmembers: A randomized controlled trial by Yara Q. Wingelaar-Jagt, Charelle Bottenheft, Wim J. Riedel and Johannes G. Ramaekers in Journal of Psychopharmacology

sj-docx-2-jop-10.1177_02698811221142568 – Supplemental material for Effects of modafinil and caffeine on night-time vigilance of air force crewmembers: A randomized controlled trial Click here for additional data file. Supplemental material, sj-docx-2-jop-10.1177_02698811221142568 for Effects of modafinil and caffeine on night-time vigilance of air force crewmembers: A randomized controlled trial by Yara Q. Wingelaar-Jagt, Charelle Bottenheft, Wim J. Riedel and Johannes G. Ramaekers in Journal of Psychopharmacology

sj-docx-3-jop-10.1177_02698811221142568 – Supplemental material for Effects of modafinil and caffeine on night-time vigilance of air force crewmembers: A randomized controlled trial Click here for additional data file. Supplemental material, sj-docx-3-jop-10.1177_02698811221142568 for Effects of modafinil and caffeine on night-time vigilance of air force crewmembers: A randomized controlled trial by Yara Q. Wingelaar-Jagt, Charelle Bottenheft, Wim J. Riedel and Johannes G. Ramaekers in Journal of Psychopharmacology
