# Supplementary material for: Effects of modafinil and caffeine on night-time vigilance of air force crewmembers: A randomized controlled trial
Source: J Psychopharmacol. 2022 Dec 14;37(2):172–80. doi: 10.1177/02698811221142568 (PMC9912306; doi:10.1177/02698811221142568)
Supplement: sj-docx-2-jop-10.1177_02698811221142568 – Supplemental material for Effects of modafinil and caffeine on night-time vigilance of air force crewmembers: A randomized controlled trial [file sj-docx-2-jop-10.1177_02698811221142568.docx]

**Supplementary Material**

A. VigTrack – Mean Reaction Time

Mauchly’s test indicated that the assumption of sphericity had been violated for the main effects of time of assessment, *χ^2^*(27) = 118.10, *p<*.001 and the interaction effect of time of assessment and treatment, *χ^2^*(104) = 213.91, *p*<.001*.* Therefore, degrees of freedom were corrected using Huynh-Feldt estimates of sphericity (ε = .40 for the main effect of time of assessment and .68 for the interaction effect of time of assessment and treatment).

B. VigTrack – Mean percentage omissions

Mauchly’s test indicated that the assumption of sphericity had been violated for the main effects of time of assessment, *χ^2^*(27) = 289.41, *p<*.001 and the interaction effect of time of assessment and treatment, *χ^2^*(104) = 375.14, *p*<.001*.* Therefore, degrees of freedom were corrected using Huynh-Feldt estimates of sphericity (ε = .22 for the main effect of time of assessment and .35 for the interaction effect of time of assessment and treatment).

C. VigTrack – Mean tracking error

Mauchly’s test indicated that the assumption of sphericity had been violated for the main effects of treatment, *χ^2^*(2) = 25.43, *p<*.001, for the main effects of time of assessment, *χ^2^*(27) = 240.74, *p<*.001 and the interaction effect of time of assessment and treatment, *χ^2^*(104) = 465.07, *p*<.001*.* Therefore, degrees of freedom were corrected using Huynh-Feldt estimates of sphericity (ε = .67 for the main effect of treatment, ε = .32 for the main effect of time of assessment and .27 for the interaction effect of time of assessment and treatment).

D. PVT – 1/ reaction time

Mauchly’s test indicated that the assumption of sphericity had been violated for the main effects of time of assessment, *χ^2^*(27) = 61.15, *p<*.001 and the interaction effect of time of assessment and treatment, *χ^2^*(104) = 185.37, *p*<.001*.* Therefore, degrees of freedom were corrected using Huynh-Feldt estimates of sphericity (ε = .66 for the main effect of time of assessment and .75 for the interaction effect of time of assessment and treatment).

E. PVT – Number of lapses

Mauchly’s test indicated that the assumption of sphericity had been violated for the main effects of time of assessment, *χ^2^*(27) = 82.51, *p<*.001 and the interaction effect of time of assessment and treatment, *χ^2^*(104) = 181.29, *p*<.001*.* Therefore, degrees of freedom were corrected using Huynh-Feldt estimates of sphericity (ε = .70 for the main effect of time of assessment and .68 for the interaction effect of time of assessment and treatment).

|  | **p-values per time of assessment** | | | | | | |
| --- | --- | --- | --- | --- | --- | --- | --- |
| Parameter | T = 0  *(0 AM)* | T = +1  *(1 AM)* | T = +2  *(2 AM)* | T = +3  *(3 AM)* | T = +4  *(4 AM)* | T = +6  *(6 AM)* | T = +8  *(8 AM)* |
| VigTrack – mean tracking error | .667 | .177 | .634 | .822 | .278 | .008* | <.001* |
| VigTrack – mean percentage omissions | .898 | .594 | .738 | .878 | .166 | .004* | .005* |
| VigTrack – mean reaction time | .256 | .498 | .857 | .086 | .007* | <.001* | .003* |
| PVT – 1/ mean reaction time | .546 | .483 | .009* | .004* | <.001* | <.001* | <.001* |
| PVT – number of lapses | .019* | .688 | .020* | .007* | <.001* | <.000* | <.000* |
| SSS | .312 | .818 | .130 | .057 | .006* | .001* | .000* |

* *p<.05*

**Table S.1.a.** P-values of the post-hoc pairwise comparisons; modafinil vs. placebo

|  | **p-values per time of assessment** | | | | | | |
| --- | --- | --- | --- | --- | --- | --- | --- |
| Parameter | T = 0  *(0 AM)* | T = +1  *(1 AM)* | T = +2  *(2 AM)* | T = +3  *(3 AM)* | T = +4  *(4 AM)* | T = +6  *(6 AM)* | T = +8  *(8 AM)* |
| VigTrack – mean tracking error | .270 | .567 | .993 | .612 | .318 | .081 | .217 |
| VigTrack – mean percentage omissions | .593 | .366 | .698 | .284 | .054 | .048* | .037* |
| VigTrack – mean reaction time | .596 | .378 | .223 | .069 | .002* | .003* | .002* |
| PVT – 1/ mean reaction time | .761 | .446 | .009* | .001* | <.001* | .001* | .003* |
| PVT – number of lapses | .534 | .069 | .003* | <.000* | <.000* | <.000* | .003* |
| SSS | .981 | .599 | .295 | .252 | .006* | .019* | .145 |

* *p<.05*

**Table S.1.b.** P-values of the post-hoc pairwise comparisons; caffeine vs. placebo

|  | **p-values per time of assessment** | | | | | | |
| --- | --- | --- | --- | --- | --- | --- | --- |
| Parameter | T = 0  *(0 AM)* | T = +1  *(1 AM)* | T = +2  *(2 AM)* | T = +3  *(3 AM)* | T = +4  *(4 AM)* | T = +6  *(6 AM)* | T = +8  *(8 AM)* |
| VigTrack – mean tracking error | .251 | .197 | .634 | .353 | .675 | .666 | .506 |
| VigTrack – mean percentage omissions | .463 | .923 | .954 | .343 | .497 | .816 | .141 |
| VigTrack – mean reaction time | .432 | .215 | .331 | .593 | .765 | .228 | .563 |
| PVT – 1/ mean reaction time | .860 | .125 | .327 | .773 | .520 | .080 | .015* |
| PVT – number of lapses | .239 | .111 | .327 | .356 | .938 | .159 | .041* |
| SSS | .259 | .360 | .869 | .094 | .732 | .191 | .006* |

* *p<.05*

**Table S.1.c.** P-values of the post-hoc pairwise comparisons; modafinil vs. caffeine
