# Supplementary figures and images for: Effects of modafinil and caffeine on night-time vigilance of air force crewmembers: A randomized controlled trial
Source: J Psychopharmacol. 2022 Dec 14;37(2):172–80. doi: 10.1177/02698811221142568 (PMC9912306; doi:10.1177/02698811221142568)

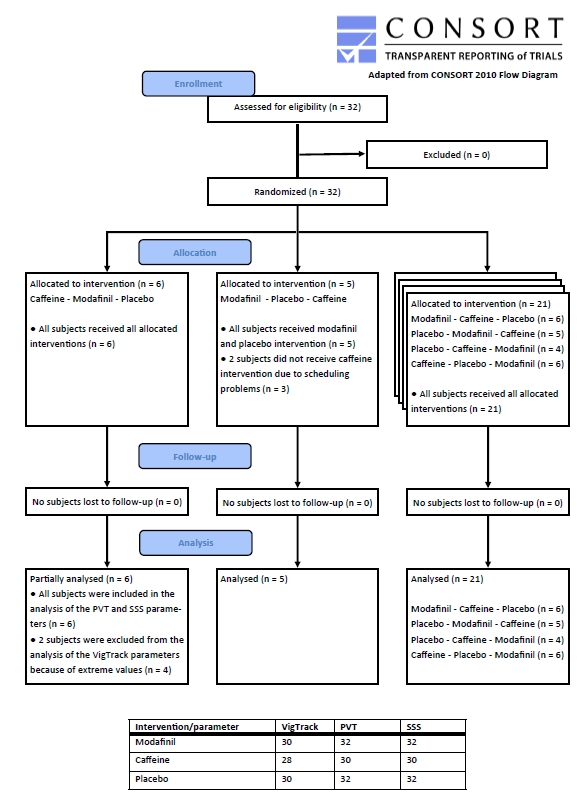

Supplement: sj-docx-3-jop-10.1177_02698811221142568 – Supplemental material for Effects of modafinil and caffeine on night-time vigilance of air force crewmembers: A randomized controlled trial [file sj-docx-3-jop-10.1177_02698811221142568.docx]
